# Increasing incidence of primary shoulder arthroplasty in Finland – a nationwide registry study

**DOI:** 10.1186/s12891-018-2150-3

**Published:** 2018-07-21

**Authors:** Jenni N. E. Harjula, Juha Paloneva, Jaason Haapakoski, Juha Kukkonen, Ville Äärimaa, the Finnish Shoulder Arthroplasty Registry Group, Pirjo Honkanen, Tapio Flinkkilä, Antti Joukainen, Konsta Pamilo, Mikko Salmela, Ville Äärimaa, Keijo Mäkelä

**Affiliations:** 1 Department of Orthopedics and Traumatology, Turku University Hospital, University of Turku, Turku, Finland; 2 Department of Surgery, Central Finland Hospital, Jyväskylä, Finland; 3 National Institute for Health and Welfare, Helsinki, Finland; 4 Department of Surgery, Division of Orthopaedic and Trauma Surgery, Satakunta Central Hospital and University of Turku, Turku, Finland

**Keywords:** Arthroplasty, Incidence, Shoulder joint, Finland

## Abstract

**Background:**

The incidence of shoulder arthroplasties is reportedly increasing and the types of arthroplasty are changing. The purpose of this study was to investigate the incidence of primary shoulder arthroplasty in Finland.

**Methods:**

We analyzed nationwide data from the Finnish Arthroplasty Register (FAR) and the Finnish National Hospital Discharge Register (NHDR) during time period 2004–2015. The primary outcome variable was the incidence of shoulder arthroplasty per 100,000 person-years stratified by age, sex and year of surgery. The secondary outcome variables were surgical indication, arthroplasty type and prosthesis model.

**Results:**

The number of primary shoulder arthroplasties was 7504 (women = 4878, men = 2625). The rate of operations increased from 6 to 15 per 100,000 person-years among men, and 11 to 26 per 100,000 person-years among women. The indication for arthroplasty was osteoarthritis in 56%, acute fracture in 21%, inflammatory arthritis in 13%, and rotator cuff arthropathy in 4% of the cases. Hemiarthroplasties accounted for 66%, total shoulder arthroplasties 8%, and reverse shoulder arthroplasties 12% of the cases, 14% of the cases was missing. During the 12-year study period the incidence of hemiarthroplasties decreased by 23% and the number of total shoulder and reverse shoulder arthroplasty increased by 500 and 4500%, respectively.

**Conclusions:**

The incidence of primary shoulder arthroplasty has increased by 160% during the study period in Finland. The incidence of hemiarthroplasties decreased while total and reverse shoulder arthroplasties increased.

**Electronic supplementary material:**

The online version of this article (10.1186/s12891-018-2150-3) contains supplementary material, which is available to authorized users.

## Background

Shoulder pain and stiffness are the third most common musculoskeletal complaints in the elderly Finnish population [1]. These symptoms may be attributable to progressive traumatic or degenerative changes in the glenohumeral joint. Eventually these symptoms can be surgically treated with shoulder arthroplasty. Generally there are two alternative surgical options for shoulder arthroplasty, an anatomic (hemi or total) or non-anatomic (reverse) arthroplasty type. The condition of the rotator cuff muscles and glenoid cartilage influence on the arthroplasty type. An anatomic arthroplasty is indicated if the rotator cuff muscles are intact.

According to registry-based studies, the incidence of shoulder arthroplasties has increased in eg. Australia, New Zealand, Denmark and United States [2–8]. Also the National Joint Registry of England, Wales, Northern Ireland and the Isle of Man has reported similar results [9]. Total anatomic and reverse shoulder arthroplasty have become increasingly common whereas the proportion of hemiarthroplasty has decreased [8]. The arthroplasty registries provide important information on country specific treatment practices and distribution of arthroplasty types. In Finland data on shoulder arthroplasties is collected in the Finnish Arthroplasty Register (FAR) founded in 1981 and also in the Finnish National Discharge Register (NHDR) founded in 1967.

This study was set out to investigate the incidence of shoulder arthroplasty and arthroplasty types in Finland after 2004. We hypothesized that the incidence of primary shoulder arthroplasty in Finland would follow the previously reported increasing international trend.

## Methods

We reviewed and combined nationwide data from FAR and NHDR during time period 2004–2015. FAR contains data on age, side, gender, domicile, type of hospital, arthroplasty component product coding and indication for operation. NHDR contains data on age, gender, domicile, type of hospital, and codes for diagnoses (according to ICD-10) and procedures (Nomesco Classification of Surgical Procedures, Finnish version). Data collection for both the NHDR and FAR is mandatory for all public and private hospitals. The validity of the NHDR has been found to be good regarding both the coverage and accuracy of data [10–12]. Specific data on the accuracy of the NHDR regarding shoulder arthroplasty are not available.

In order to capture all cases and assess the coverage of FAR reporting, the data in FAR and NHDR on primary shoulder arthroplasties were combined and thereafter the duplicates were removed. The indications for primary shoulder arthroplasty were categorized according to Nordic Arthroplasty Registry Association (NARA) [8] as: osteoarthritis, fracture sequelae, inflammatory arthritis, rotator cuff arthropathy, acute fracture, others and missing (Additional file 1). The FAR diagnosis was used as the primary data source. If the FAR diagnosis was not known (else or missing), we then used the combination of both FAR and NHDR diagnosis. The FAR data collecting paper is designed for hips and knees. Thus, the indications for surgery are rheumatoid arthritis, other arthritis, primary osteoarthrosis, congenital hip luxation, other illness, the change of the prothesis, the removal of the prothesis, secondary arthrosis, the removal of previous prothesis and other revision. For shoulders, the suitable indications for surgery are rheumatoid arthritis, other arthritis (else arthritis in the FAR column), primary osteoarthrosis, other illness (else in the FAR column) and secondary arthrosis. In some cases the indication for surgery was unknown and is marked as missing inthe FAR column. The data from the FAR data collecting papers were then supplemented with NHDR data. The most commonly used ICD-10 diagnosis have been marked in the NHDR column in Additional file 1. After combining FAR and NHDR databases, the NARA diagnosis was synthesized. The NARA diagnosis is based on NHDR database if the indication in FAR column is “Else” or” missing”.

Arthroplasty type was categorized as hemi-, total-, reverse shoulder arthroplasty or missing, and determined by combining the data on the type of stem and glenoid component recorded in FAR, and the NHDR procedure code (NBB10 for hemi and NBB20 for total/reverse). In case of discrepancy between FAR and NDHR, the arthroplasty type was recorded based on FAR. Arthroplasty type in NBB20 procedures, without data on prosthesis model, was categorized as missing.

### Statistics

We used national population data obtained from Statistics Finland [13] to calculate the incidence of primary shoulder arthroplasty. The primary outcome variable in this study was the incidence of shoulder arthroplasty per 100,000 person-years, which was analyzed with stratification by age, sex and year of surgery. The secondary outcome variables were indication, arthroplasty type and prosthesis model. Incidence rates were calculated using the annual adult population size, ranging from 4.1 (year 2004) to 4.4 million (year 2015) during the study period. Incidence of the operations per 100,000 person-years was calculated by dividing the annual number of procedures by the size of the population aged ≥18 years in the end of the year in question, multipilied by 100,000. The incidence was based on the size of the entire population of persons ≥18 years old in Finland rather than cohort –based estimates. Accordingly, confidence intervals were not calculated.

Poisson regression was used to analyze the operations per 100,000 person years. Two separate models were fitted; the first one included sex and year and the second one included age group and year. The year was considered as a continuous variable due to somewhat linear growth over years in both models. Also, the interactions of sex and year as well as age group and year were evaluated. However, the interactions were not included in the final models as they were not statistically significant. The results are quantified using relative risks with 95% confidence intervals (95% CI). *P*-values less than 0.05 were considered as statistically significant. Statistical analyses were carried out using SAS system for Windows, Version 9.4 (SAS Institute Inc., Cary, NC, USA).

### Ethics

Ethics approval was granted by Finland’s National Institute for Health and Wellness (Dnro THL/1743/5.05.00/2014).

## Results

The number of primary shoulder arthroplasties during the 12-year study period was 4618 (women = 2976, men = 1641, missing = 1) in FAR and 7504 (women = 4878, men = 2625, missing = 1) in NHDR. The FAR coverage is shown in Table 1. 96% of the data in FAR matched the data from NHDR in terms of indication and 86% in terms of arthroplasty type (hemi or total). Of the 2886 arthroplasties not reported in FAR, 2039 (71%) had hemiarthroplasty coding and the rest 29% were missing. The diagnosis was osteoarthritis in 1332 (46%), fracture sequelae in 35 (1%), inflammatory arthritis in 201 (7%), rotator cuff arthropathy in 134 (5%), acute fracture 1054 (37%), others 80 (2%) and missing in 50 (2%) of the 2886 arthroplasties not reported on FAR.

**Table 1:** The Finnish Arthroplasty register coverage compared to the Finnish National Hospital Discharge Register regarding shoulder arthroplasties. The NHDR coverage compared to the FAR was 100%

YEAR	NHDR	FAR	FAR COVERAGE %
2004	357	265	74
2005	400	252	63
2006	445	303	68
2007	502	353	70
2008	570	391	69
2009	624	409	66
2010	650	409	63
2011	780	507	65
2012	790	508	64
2013	713	409	57
2014	761	401	53
2015	912	411	45
TOTAL	7504	4618	62

*FAR* the Finnish Arthroplasty register, *NHDR* the Finnish National Hospital Discharge Register

**Fig. 1:**
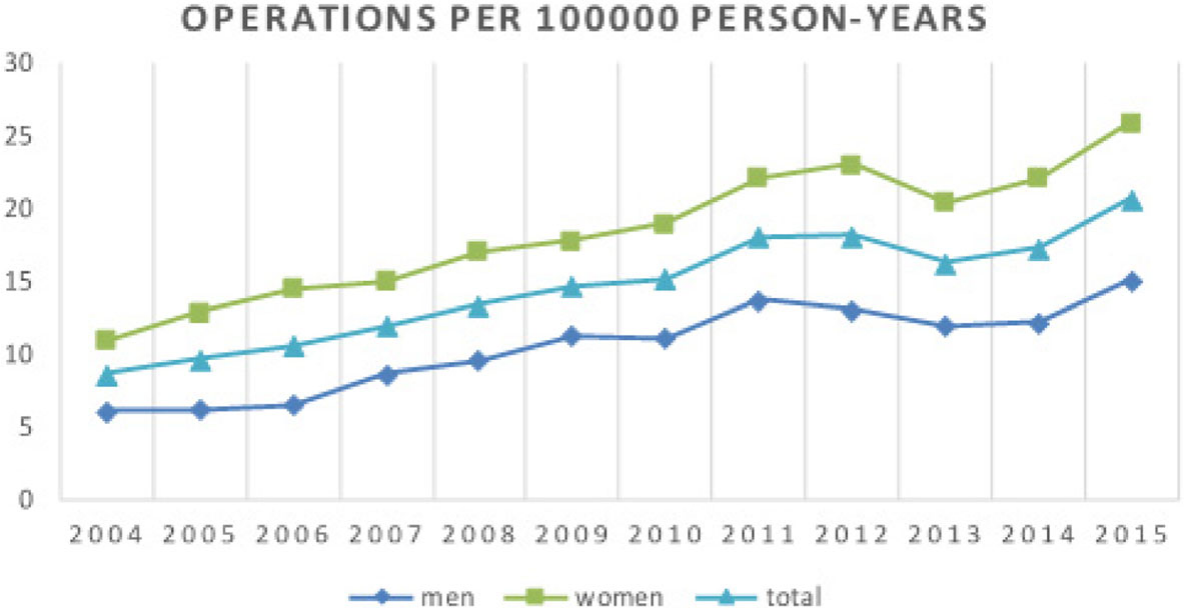
The incidence per 100,000 person-years of primary shoulder arthroplasty in the Finnish adult population by year

**Fig. 2:**
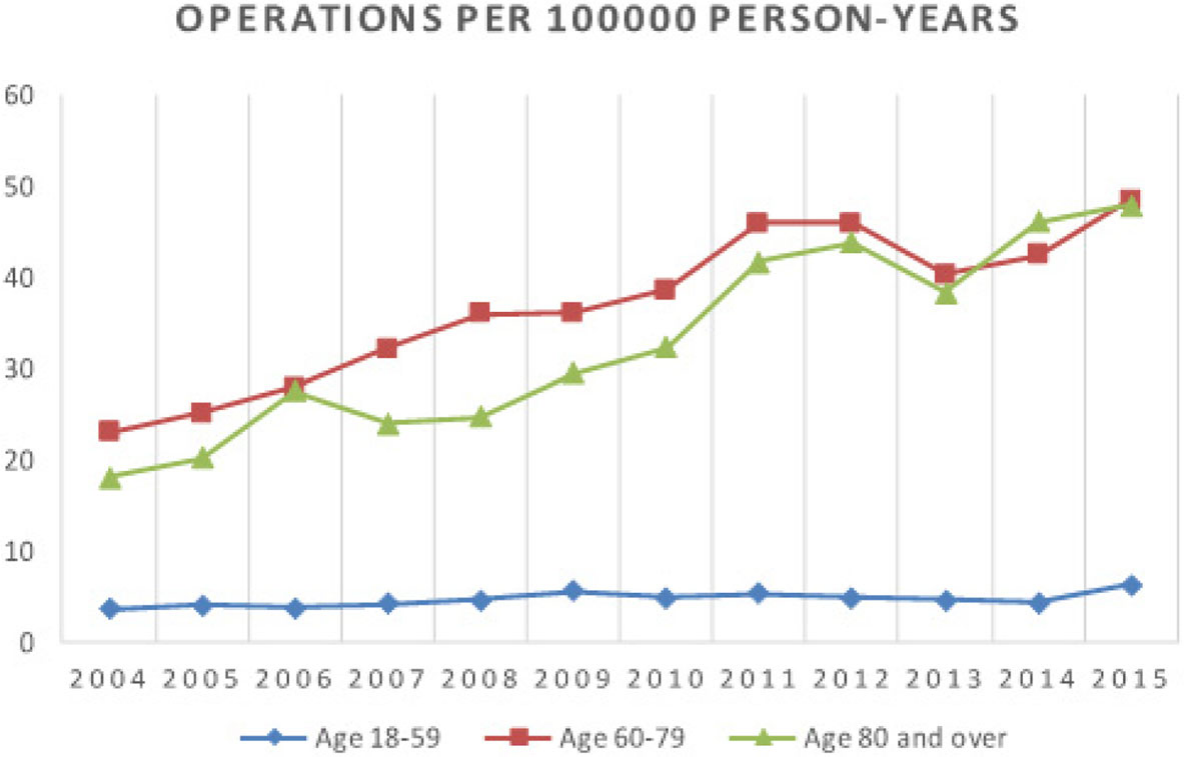
The incidence of primary shoulder arthroplasty among patients in different age groups in Finland by year

The incidence of annual primary shoulder arthroplasty for each year during the study period is presented in Fig. 1. From 2004 to 2015 the rate of operations increased from 9 to 21 per 100,000 person-years altogether and 6 to 15 per 100,000 person-years among men, and from 11 to 26 for women. The incidence rates have increased linearly and statistically significantly over the years (*p* < 0.001). The number of operations increased 7% every year (RR 1.07, 95% CI: 1.04 to 1.11) which means that the number of operations have increased about doubled during 10 years (RR 2.01, 95% CI 1.48 to 2.74). Women had significantly more operations than men (*p* < 0.001, RR 1.76, 95% CI: 1.41 to 2.19). Over the study period, the mean patient age at the time of primary operation was 67 years (range 20–96) (men 67, women 67). The majority (64%) of patients were treated between ages 60–79 years. There was a statistically significant difference between the age-groups in the incidence of operations (*p* < 0.001). The age group 60–79 was 7.78 (95% CI 5.90 to 10.25) times as likely to have an operation than the group of 18–59 years (*p* < 0.001) and 80+ group was 6.94 (95% CI 5.25 to 9.16) as likely to have an operation than the group of 18–59 years (p < 0.001). There was no statistically significant difference between age groups 60–79 and 80+ (*p* = 0.097, RR 1.12, 95% CI 0.98 to 1.28). The incidence of primary shoulder arthroplasty among patients in different age groups is presented in Fig. 2.

**Fig. 3:**
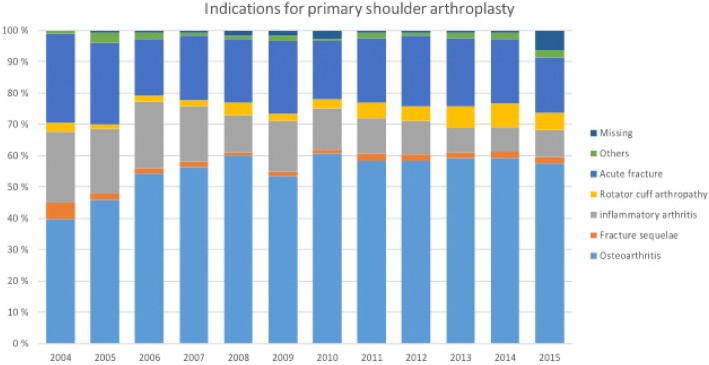
Reported indications for primary shoulder arthroplasty during the study period. The number of annual indications are presented in Additional file 3

The most common indication for primary shoulder arthroplasty was osteoarthritis in 56% of the cases (Fig. 3). Acute fracture was the second (21%) and inflammatory arthritis the third most common (13%) indication. Rotator cuff arthropathy was recorded in 4% and fracture sequealae in 2% of cases. In 2% of cases the indication was other and in 2% of the cases the indication was missing.

Arthroplasty type could be identified in 86% of the cases. Hemiarthroplasties accounted for the majority (66%), total shoulder arthroplasties in 8%, and reverse shoulder arthroplasties in 12% of the cases. The most common arthroplasty type for osteoarthritis was hemiarthroplasty (65%). Similarly, a hemiarthroplasty was recorded in 84% of the patients with an acute fracture diagnosis. The most common arthroplasty type for inflammatory arthritis was also hemiarthroplasty in 60% of cases. The arthroplasty types and diagnosis are presented in Table 2.

**Table 2:** The number and percentage of primary arthroplasty types, together with patient mean age and gender in categorized indications during the study period

	Osteo-arthritis	Fracture sequelae	Inflammatory arthritis	Rotator cuff arthropathy	Acute fracture	Others	Missing	Total
Hemiarthroplasty								
n	2728	79	580	85	1328	81	48	4929
(%)	55	2	11	2	27	2	1	100
Mean age	65.8	61.2	63.4	65.5	69.9	63.5	66.3	66.5
(women %)	53	56	80	41	75	62	73	62
Total arthroplasty	497	10	76	3	24	1	2	613
	81	2	12	1	4	0	0	100
	67.8	65.3	66.8	72.3	70.1	76	77.5	67.8
	60	70	80	100	79	100	100	63
Reverse arthroplasty	396	35	214	134	102	12	35	928
	43	4	23	14	11	1	4	100
	73.7	68.7	70.9	74.8	73.1	75.4	74.4	73.0
	71	89	86	69	76	83	74	76
Missing	613	21	101	103	129	27	40	1038
	59	2	10	10	12	3	4	100
	69.0	71.5	66.5	73.2	72.6	64.6	67.1	69.5
	63	76	81	76	79	59	75	68
Total	4234	145	971	325	1583	121	125	7504
	53	2	13	4	21	2	2	100
	67.2	64.8	65.6	71.8	70.3	65.0	69.0	67.8
	57	68	81	64	76	64	74	65

**Fig. 4:**
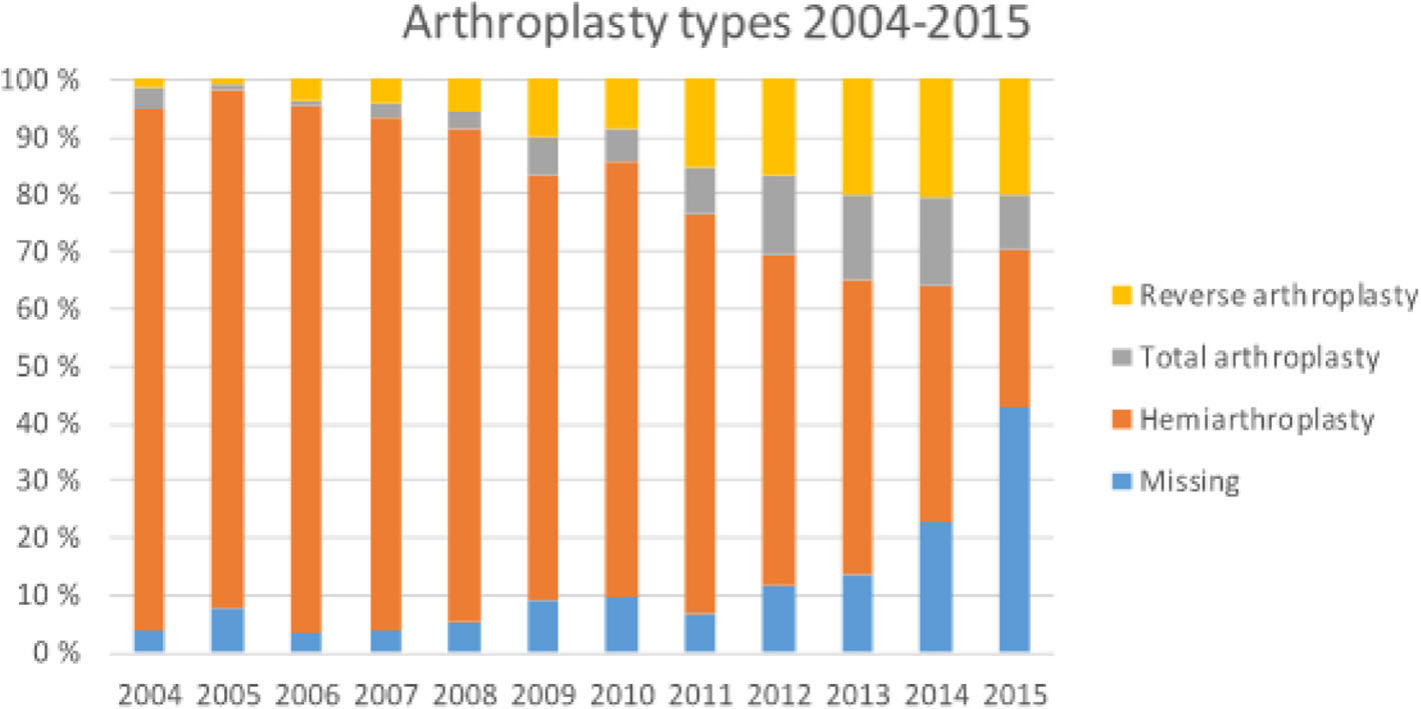
The change in arthroplasty types during the study period. Y-axis indicates the annual number. The number of annual arthroplasties are presented in Additional file 4

The distribution of arthroplasty types is shown in Fig. 4. During the study period the total number of hemiarthroplasty decreased 23% and the number of total shoulder arthroplasty and reverse shoulder arthroplasty increased by 500 and 4500% 46, respectively.

**Fig. 5:**
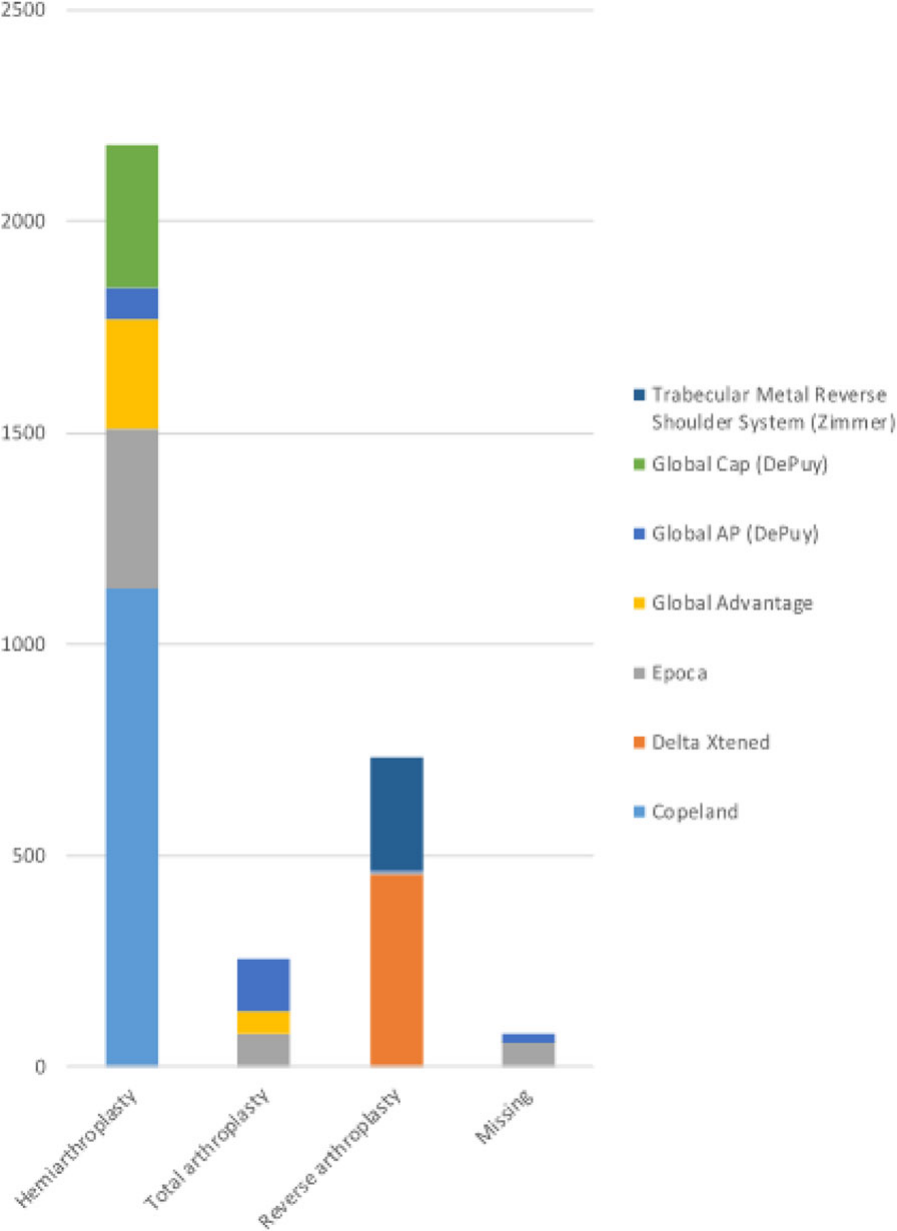
The most common reported prosthesis models (= each used in over 200 cases) for primary shoulder arthroplasty in Finland during the study period

Prosthesis model could be identified in 59% of the cases including 32 different models during the study period. The most commonly reported prosthesis models are shown in Fig. 5. Copeland was the most common hemiarthroplasty (*n* = 1131, 23%), Global AP (DePuy) the most common total shoulder (*n* = 126, 9%) and Delta Xtend the most common reverse (*n* = 459, 49%) arthroplasty model.

## Discussion

The results of our study corroborate the hypothesis and show a 160% increase in primary shoulder arthroplasty during the study period between 2004 and 2015. This is in line with the previously published reports [2–9]. Accordingly, the proportion of hemiarthroplasties has decreased and total shoulder and reverse arthroplasty increased in Finland.

In our study, the incidence of shoulder arthroplasty was 21 per 100,000 at the end of the study period. This is higher than reported from the Nordic Arthroplasty Register Association (Denmark 13.3/100,000, Norway 7.1/100,000 and Sweden 9.1/100,000) [8]. The number of shoulder arthroplasties is rapidly increasing internationally. In Australia there was a 89% increase from 2008 to 2015 and at the same time the proportion of total shoulder replacements increased from 58 to 82% [5]. In the National Joint Registry of England, Wales, Northern Ireland and the Isle of Man the number of primary shoulder arthroplasties has increased 118% from year 2012 to 2015 [9]. Increasing awareness of shoulder arthroplasty by both patients and physicians together with a readily available public health care may add to the detected current higher incidence of shoulder arthroplasties in Finland. Similarly, the reported incidences of other types of surgery such as total hip and knee arthroplasty, rotator cuff reconstruction and knee arthroscopy, have been relatively high in Finland compared to reports from other countries [14–18]. The mean age of the primary shoulder arthroplasty patients at the end of the study period was 67 years and it is comparable to other studies from the US as well as Nordic and British populations [3, 6, 8, 9]. Similarly to other studies, we found that the increasing majority of patients were women [6–9].

The most common and increasingly reported indication for primary shoulder arthroplasty in our cohort was osteoarthritis. The second most common was fracture that maintained a constant incidence throughout the study period. The third and fourth most common indications, inflammatory arthritis and rotator cuff arthropathy, were infrequently reported similarly to the Nordic Arthroplasty Register Association [8]. Inflammatory arthritis requiring surgical intervention is becoming a proportionally rare condition potentially due to advanced medical treatment options [19]. However, rotator cuff arthropathy is a common age-related condition [20] and the small registered number is likely due to missing diagnostic coding in FAR. In our study this indication was given only if both osteoarthritis and/or rotator cuff disease diagnosis were recorded. Therefore, it is likely that some patients with only osteoarthritis diagnosis, had in fact rotator cuff arthropathy.

The prevalence of rotator cuff tears increases with advancing age [21]. As the oldest age group in the population grows in Finland it could be anticipated that an increasing number of patients seek treatment not only for osteoarthritis but also for rotator cuff disease. It has been shown that rotator cuff tears are increasingly often treated surgically in the elderly population [17]. These observations may be associated with the increasing incidence of reverse shoulder arthroplasty. However, the absolute and proportional number of reverse shoulder prosthesis is still low in Finland compared to other Nordic countries [8]. Nevertheless, the simultaneous decline of hemiarthroplasties and increase of total shoulder arthroplasties is noteworthy in our cohort. This is in line with the Kaiser Permanent Shoulder Arthroplasty Registry [6] and is associated with reports on superior clinical outcome of total shoulder arthroplasty compared to hemiarthroplasty [22–24].

The vast number of different prosthesis models represents non-uniform and sporadic shoulder arthroplasty practices in Finland. Copeland was the most commonly reported implant during the study period. This resurfacing implant has been associated with inferior results and high revision rates [25]. On the other hand the most common total shoulder arthroplasty Global AP and reverse shoulder arthroplasty Delta Xtend have been associated good survival on Australian Shoulder Arthroplasty registry [5]. The observed increase in total and reverse shoulder arthroplasty may be explained by the reportedly good results and survival of TSA and RSA [5].

The Finnish Arthroplasty Register (FAR) data collecting paper forms were originally designed for hip and knee arthroplasty and therefore the applicability for recording shoulder arthroplasties has been limited. The shoulder arthroplasty type coding could be reliably identified in the FAR only after 2004 after which time point we extracted the studied data. A major limitation of FAR concerning shoulder arthroplasty surgery is the increasingly poor coverage despite its national and obligatory nature. Therefore, the prosthesis model could be determined in only 59% of the cases. Also the information about the resurfacing procedure or stemmed and stemless humeral implants is limited. FAR is currently under renewal and the paper format will be replaced by shoulder specific electronic online software in the future. A second limitation is the insufficient coding for both diagnosis and operations. ICD-10 or Nomesco do not contain a universal coding for rotator cuff arthropathy, nor for reverse shoulder arthroplasty – this information was gathered by combining data from FAR and NHDR and the data may contain human error. The third limitation is the lack of patient reported outcome measures. The future Finnish shoulder arthroplasty registry will contain a shoulder specific data set including information on eg. imaging data and PROM outcomes.

The main strength of our study is the combined information from FAR and NHDR registries. The reported information in the FAR and NHDR was 90% matching. Most of the unreported data in FAR was coded as hemiarthroplasty in NHDR and enabled reliable arthroplasty typing in 89% of the cases. The data from the NHDR have been previously shown to have good validity regarding both coverage and accuracy [26, 27]. The NHDR covers the entire country, including both public and private hospitals. The total primary shoulder arthroplasty incidence may therefore be considered representative.

## Conclusion

The incidence of shoulder arthroplasty in Finland has increased almost linearly by 260% during the 12-year study period. We may therefore expect a further increase in the rate of shoulder arthroplasties, and moreover revisions, in the future. The poor coverage of FAR is alarming. New emerging treatment practices, such as reverse shoulder arthroplasty with various prosthesis designs, require meticulous tracking and communication of the outcomes and complications. Functional arthroplasty registries with obligatory input and output of comprehensive data, may provide an important mechanism for this in the future.

## Additional files

Additional file 1: The protocol for combining the FAR and NHDR diagnostic data according to NARA diagnosis categories. The NHDR diagnoses for other osteoarthritis, other fracture sequelae, other inflammatory arthritis, other fracture are presented in Additional file 2. (DOCX 13 kb)

Additional file 2: The NHDR diagnoses for other osteoarthritis, other fracture sequelae, other inflammatory arthritis, other fracture. (DOCX 15 kb)

Additional file 3: The number of annual indications for primary shoulder arthroplasty. (DOCX 14 kb)

Additional file 4: The number of annual arthroplasties for primary shoulder arthroplasty. (DOCX 13 kb)

## Abbreviations

FAR: The Finnish Arthroplasty Register
NHDR: The Finnish National Hospital Discharge Register
NARA: Nordic Arthroplasty Registry Association
