# Supplementary material for: Increasing incidence of primary shoulder arthroplasty in Finland – a nationwide registry study
Source: BMC Musculoskelet Disord. 2018 Jul 21;19:245. doi: 10.1186/s12891-018-2150-3 (PMC6054850; doi:10.1186/s12891-018-2150-3)
Supplement: Supplementary file 1 — The protocol for combining the FAR and NHDR diagnostic data according to NARA diagnosis categories. The NHDR diagnoses for other osteoarthritis, other fracture sequelae, other inflammatory arthritis, other fracture are presented in Additional file 2. (DOCX 13 kb) [file 12891_2018_2150_MOESM1_ESM.docx]

| FAR | NHDR | NARA (n=7504) |
| --- | --- | --- |
| primary arthrosis (n=2598) OR  secondary arthrosis (n=252) OR  else (n=52) OR  missing (n=1332) | M19.0 Primary arthrosis (n=3040)  M19.1 Posttraumatic arthrosis (n=405)  Other osteoarthritis (n=223) | Osteoarthritis (n=4234) |
| else (n=110) OR  missing (n=35) | M84.1 **Nonunion of fracture(**n=33)  Other fracture sequelae (n=43) | Fracture sequelae (n=145) |
| rheumatoid arthritis (n=697) OR  else arthritis (n=62) OR  else (n=11) OR missing (n=201) | M05.8 Seropositive rheumatoid arthritis (n=605)  M06.0 Seronegative rheumatoid arthritis (n=60)  Other inflammatory arthritis (n=71) | Inflammatory arthritis (n=971) |
| primary arthrosis (n=97) OR  secondary arthrosis (n=22) OR  else (n=72) OR  missing (n=134) | M75.1 Rotator cuff arthropathy (n=278)  S46.0 **Injury of tendon of the rotator cuff of shoulder** (n=14) | Rotator cuff arthropathy (n=325) |
| else (n=529) OR  missing (n=1054) | S42.2 Proximal humerus fracture (n=1412)  Other fracture (n=65) | Acute fracture (n=1583) |
| else (n=41) OR  missing (n=80) | Tumors (n=15)  Instability (n=45)  Osteonecrosis (n=11)  Others (n=36) | Others (n=121) |
| else (n=74) OR  missing (n=51) |  | Missing (n=125) |
