# Supplementary material for: Increasing incidence of primary shoulder arthroplasty in Finland – a nationwide registry study
Source: BMC Musculoskelet Disord. 2018 Jul 21;19:245. doi: 10.1186/s12891-018-2150-3 (PMC6054850; doi:10.1186/s12891-018-2150-3)
Supplement: Supplementary file 2 — The NHDR diagnoses for other osteoarthritis, other fracture sequelae, other inflammatory arthritis, other fracture. (DOCX 15 kb) [file 12891_2018_2150_MOESM2_ESM.docx]

|  | NHDR |
| --- | --- |
| Other osteoarthritis | M19.8 **Other specified arthrosis**  M19.2 **Other secondary arthrosis**  M19.9 **Arthrosis, unspecified**  M15.0 **Primary generalized (osteo)arthrosis** |
| Other fracture sequelae | M84.0 **Malunion of fracture**  T92.1 **Sequelae of fracture of arm**  **M84.2 Delayed union of fracture** |
| Other inflammatory arthritis | M08.3 **Juvenile polyarthritis (seronegative)**  M79.0 **Rheumatism, unspecified**  M07.3 **Other psoriatic arthropathies**  M05.9 **Seropositive rheumatoid arthritis, unspecified**  M13.9 **Arthritis, unspecified**  M45 **Ankylosing spondylitis**  M08.0 **Juvenile rheumatoid arthritis**  M13.1 **Monoarthritis, not elsewhere classified**  **M24.1 Other articular cartilage disorders** |
| Other fracture | S42.4 **Fracture of lower end of humerus**  S42.9 **Fracture of shoulder girdle, part unspecified**  S42.1 **Fracture of scapula**  S42.3 **Fracture of shaft of humerus** |
| Others | Tumors   - D16.1 **Benign neoplasm of short bones of upper limb** - C40 **Malignant neoplasm of bone and articular cartilage of limbs** - D48.0 **Neoplasm of uncertain or unknown behaviour of other and unspecified sites, Bone and articular cartilage**   Instability   - T92.3 **Sequelae of dislocation, sprain and strain of upper limb** - T92.8 **Sequelae of other specified injuries of upper limb** - S43.0 **Dislocation of shoulder joint** - M24.4 **Recurrent dislocation and subluxation of joint**   Osteonecrosis   - M87.0 **Idiopathic aseptic necrosis of bone** - M87.9 **Osteonecrosis, unspecified** - M87.2 **Osteonecrosis due to previous trauma** - M87.3 **Other secondary osteonecrosis**   Others   - S42.0 **Fracture of clavicle** - M90.7 **Fracture of bone in neoplastic disease** - **T92.8 Sequelae of other specified injuries of upper limb** - **S49.9 Unspecified injury of shoulder and upper arm** - **M90.6 Osteitis deformans in neoplastic disease** - **M84.4 Pathological fracture, not elsewhere classified** |
