# Supplementary material for: Increasing incidence of primary shoulder arthroplasty in Finland – a nationwide registry study
Source: BMC Musculoskelet Disord. 2018 Jul 21;19:245. doi: 10.1186/s12891-018-2150-3 (PMC6054850; doi:10.1186/s12891-018-2150-3)
Supplement: Supplementary file 3 — The number of annual indications for primary shoulder arthroplasty. (DOCX 14 kb) [file 12891_2018_2150_MOESM3_ESM.docx]

| Year | Osteo-arthritis | Fracture sequelae | inflammatory arthritis | Rotator cuff arthropathy | Acute fracture | Others | Missing | Total |
| --- | --- | --- | --- | --- | --- | --- | --- | --- |
| 2004 | 142 | 19 | 80 | 11 | 101 | 3 | 1 | 357 |
| 2005 | 184 | 8 | 82 | 6 | 104 | 13 | 3 | 400 |
| 2006 | 241 | 8 | 95 | 8 | 81 | 9 | 3 | 445 |
| 2007 | 283 | 8 | 90 | 10 | 103 | 5 | 3 | 502 |
| 2008 | 343 | 5 | 67 | 24 | 115 | 7 | 9 | 570 |
| 2009 | 333 | 10 | 101 | 15 | 144 | 11 | 10 | 624 |
| 2010 | 395 | 6 | 88 | 19 | 121 | 3 | 18 | 650 |
| 2011 | 454 | 19 | 89 | 37 | 161 | 15 | 5 | 780 |
| 2012 | 460 | 17 | 84 | 37 | 180 | 6 | 6 | 790 |
| 2013 | 423 | 12 | 56 | 50 | 155 | 11 | 6 | 713 |
| 2014 | 451 | 14 | 61 | 58 | 156 | 16 | 5 | 761 |
| 2015 | 525 | 19 | 78 | 50 | 162 | 22 | 56 | 912 |
| **Total** | **4234** | **145** | **971** | **325** | **1583** | **121** | **125** | **7504** |
