# Supplementary material for: Increasing incidence of primary shoulder arthroplasty in Finland – a nationwide registry study
Source: BMC Musculoskelet Disord. 2018 Jul 21;19:245. doi: 10.1186/s12891-018-2150-3 (PMC6054850; doi:10.1186/s12891-018-2150-3)
Supplement: Supplementary file 4 — The number of annual arthroplasties for primary shoulder arthroplasty. (DOCX 13 kb) [file 12891_2018_2150_MOESM4_ESM.docx]

| Year | Hemiarthroplasty | Total arthroplasty | Reverse arthroplasty | Missing | Total |
| --- | --- | --- | --- | --- | --- |
| 2004 | 325 | 14 | 4 | 14 | 357 |
| 2005 | 362 | 4 | 3 | 31 | 400 |
| 2006 | 409 | 5 | 16 | 15 | 445 |
| 2007 | 450 | 13 | 20 | 19 | 502 |
| 2008 | 491 | 16 | 33 | 30 | 570 |
| 2009 | 463 | 44 | 61 | 56 | 624 |
| 2010 | 493 | 37 | 56 | 64 | 650 |
| 2011 | 548 | 61 | 120 | 51 | 780 |
| 2012 | 458 | 110 | 132 | 90 | 790 |
| 2013 | 367 | 105 | 143 | 98 | 713 |
| 2014 | 312 | 119 | 156 | 174 | 761 |
| 2015 | 251 | 85 | 184 | 392 | 912 |
| **Total** | **4929** | **613** | **928** | **1034** | **7504** |
